# Path Planning for Multi-Arm Manipulators Using Deep Reinforcement Learning: Soft Actor–Critic with Hindsight Experience Replay

**DOI:** 10.3390/s20205911

**Published:** 2020-10-19

**Authors:** Evan Prianto, MyeongSeop Kim, Jae-Han Park, Ji-Hun Bae, Jung-Su Kim

**Affiliations:** 1 Research Center for Electrical and Information Technology, Department of Electrical and Information Engineering, Seoul National University of Science and Technology, Seoul 01811, Korea; evanprianto.el@gmail.com (E.P.); kimmyungsup57@gmail.com (M.K.); 2 Applied Robot R&D Department, Korea Institute of Industrial Technology (KITECH), Ansan 15588, Korea; hans1024@kitech.re.kr (J.-H.P.); joseph@kitech.re.kr (J.-H.B.)

**Keywords:** path planning, multi-arm manipulators, reinforcement learning, soft actor-critic (SAC), hindsight experience replay (HER), collision avoidance

## Abstract

Since path planning for multi-arm manipulators is a complicated high-dimensional problem, effective and fast path generation is not easy for the arbitrarily given start and goal locations of the end effector. Especially, when it comes to deep reinforcement learning-based path planning, high-dimensionality makes it difficult for existing reinforcement learning-based methods to have efficient exploration which is crucial for successful training. The recently proposed soft actor–critic (SAC) is well known to have good exploration ability due to the use of the entropy term in the objective function. Motivated by this, in this paper, a SAC-based path planning algorithm is proposed. The hindsight experience replay (HER) is also employed for sample efficiency and configuration space augmentation is used in order to deal with complicated configuration space of the multi-arms. To show the effectiveness of the proposed algorithm, both simulation and experiment results are given. By comparing with existing results, it is demonstrated that the proposed method outperforms the existing results.

## 1. Introduction

In the Industry 4.0 era, one of the important elements in the manufacturing industry for a smart factory is automation via collaboration of robot manipulators, and the manufacturing industry has been less affected by human workforce [1]. Especially, multi-arm manipulators have been drawing more attention due to its increasing application such as assembly line in a factory, space research, customer service, exploration, even rescue mission [2]. Hence, it is utmost important to improve the efficiency of the operation of the multi-arm manipulator.

### 1.1. Motivation

For manipulators in manufacturing industry, when a specific task (e.g., moving an object) is given, human experts find manually a collision-free path from the start and goal locations for the end-effector in order to perform the task and teach the path to the manipulator. Hence, if the start and goal locations of the end-effector vary owing to task change, this procedure has to be repeated. Moreover, in the case of multi-arm manipulators, such a procedure becomes much more difficult [3,4]. Hence, it is important to make such a procedure carried out automatically. In addition to this, for the purpose of making the whole manufacturing process efficient and optimal, the multi-arm manipulator has to learn the optimal (i.e., shortest) path rather than a feasible path when arbitrary start and goal locations are given   [5,6].

### 1.2. Background and Related Work

A representative of path planning for multi-arm manipulators is the sampling-based algorithm which computes the path after building a graph using sampled points of the workspace [7]. The examples of sampling-based algorithms are fast marching method (FMM) [8], probabilistic road map (PRM) method [9], and rapid exploring random trees (RRT) method [10,11,12,13]. In PRM, the Dijkstra algorithm is applied to find the shortest paths from the constructed graph [14,15]. The path generated by the sampling-based method may not be the optimal path since the resulting path is heavily dependent on the sampling methods. The other approach to solve the path planning problem for multi-arm manipulators is to use the artificial potential function to create a motion equation that can guide the robot arm to the goal point [16,17]. By computing the gradient of the equation, the direction of the optimal path can be attained [18]. However, it can be trapped in the local minimum of the potential field and fail to find the right path [19]. This situation can be worse if the path planning problem is high-dimensional. Note that the path planning under consideration is complicated and high-dimensional by nature. Due to this reason, an effective path planning algorithm for multi-arm manipulators has to be developed. In the literature about path planning, there are already some deep learning-based approaches implemented for robot applications such as mobile manipulation [20,21], unmanned ship [22] and even for multi-mobile robot [23]. These imply that deep learning-based approach can be promising in path planning for single arm manipulator [24] and also for multi-arm manipulators [25]. Especially, the focus of this paper is placed on devising a deep reinforcement learning-based path planning algorithm for multi-arm manipulators [26].

There are mainly three difficulties in reinforcement learning-based path planning for the multi-arm manipulator. First, in order to design a path planning algorithm, although it is essential to deal with the configuration space properly, it is not easy to define the configuration space which consists of collision-free space and collision space for the multi-arm manipulator since there are multiple arms, which makes the problem nontrivial. Second, when reinforcement learning-based path planning is designed, efficient exploration is indispensable to find the shortest path. However, since path planning for the multi-arm manipulator is a high-dimensional problem, existing reinforcement learning-based path planning suffers from poor exploration performance, which makes the resulting path planning lead to a suboptimal solution (i.e., not the shortest path). Third, when path planning is designed using reinforcement learning, the agent can get the reward in a sparse manner. In other words, when arbitrary start and goal locations are given for the reinforcement learning-based path planning, there can be many cases where the agent fails to find the shortest path due to physical limitations or high dimensionality of the configuration space. In this case, the agent can not get the reward contributing to training.

### 1.3. Proposed Solution

In this paper, a recently reported policy gradient type reinforcement learning algorithm called SAC (Soft Actor-Critic)-based path planning is proposed to overcome the previously described difficulties  [27]. To this end, the configuration spaces of each arm are augmented as if the whole multi-arm manipulator is viewed as a virtual single-arm manipulator whose configuration space has higher dimension. Since SAC is known to show superior exploration performance due to the entropy term in the objective function, this paper proposes how to apply SAC to designing of a path planning algorithm. In order to overcome the sparse reward problem, HER (Hindsight Experience Replay) [28] is employed to deal with generated episodes efficiently in training. Note that HER can convert the episodes without reward to episodes with reward by changing the target location.

The performance of the proposed SAC-based path planning is validated not only simulation but also experiment using two real open manipulators. The results show that the proposed method finds a shorter and smoother path for most scenarios due to enhanced exploration performance by SAC, and outperforms over the existing results such as PRM [29] and TD3 (Twin Delayed Deep Deterministic Policy Gradient)-based path planning [30].

## 2. Problem Setup and Preliminaries

### 2.1. Sampling-Based Path Planning for Robot Manipulator and Configuration Space

In sampling-based path planning for robot manipulators, the space of possible joint angles is referred to as configuration space Q (also called joint space) and is a subset of *n*-dimensional vector space $R^{n}$ where *n* is the number of the joints of the robot manipulator. A point in the configuration space Q corresponds to values of joint angles of a robot manipulator [6,31,32]. The configuration space is comprised of two subsets: $Q_{\mathrm{collide}}$ and $Q_{\mathrm{free}}$. The robot manipulator does not collide with any obstacles or itself if its joint angles belong to $Q_{\mathrm{free}}$. If the values of the joint angles are in $Q_{\mathrm{collide}}$, it means that there is a collision between the robot manipulator and obstacles or itself.

For sampling-based path planning, random sampling for the continuous $Q_{\mathrm{free}}$ is done in order to obtain its discrete representation. Then, the discrete representation is modeled as a connected graph. From this construction, the nodes in the graph imply admissible configurations of the robot manipulators, and feasible paths between any two configurations are represented by connected edges in the graph.

For the purpose of describing the path planning problem, let ${\bar{q}}_{t}\in Q\in R^{n}$ denote the values of the joint angles of the manipulator. Also, let *T* represent the maximum number of the iteration in any path planning algorithm. This means that the algorithm either finds the shortest path successfully within *T* iterations for a given starting configuration ${\bar{q}}_{\mathrm{init}}\in Q_{\mathrm{free}}$ and goal configuration ${\bar{q}}_{\mathrm{goal}}\in Q_{\mathrm{free}}$, or decides that it is not possible to compute the shortest path for those configurations. With these definitions, when ${\bar{q}}_{\mathrm{init}}\in Q_{\mathrm{free}}$ and ${\bar{q}}_{\mathrm{goal}}\in Q_{\mathrm{free}}$ are given, path planning is to compute a valid continuous and shortest path on the graph linking ${\bar{q}}_{\mathrm{init}}$ and ${\bar{q}}_{\mathrm{goal}}$. Since the sampled nodes of $Q_{\mathrm{free}}$ are modeled as a connected graph, there always exists such a shortest path between any two nodes in $Q_{\mathrm{free}}$. Note that the path means the sequence of the state ${\bar{q}}_{t}$ such that ${\bar{q}}_{0}={\bar{q}}_{\mathrm{init}},\;\cdots ,\;{\bar{q}}_{T_{1}}={\bar{q}}_{\mathrm{goal}}$ with $T_{1}\leq T$, and both the sequence $\left\{{\bar{q}}_{0},\cdots ,{\bar{q}}_{T_{1}}\right\}$ and the line segment connecting any two consecutive state ${\bar{q}}_{t}$ and ${\bar{q}}_{t+1}$ belonging to $\left\{{\bar{q}}_{0},\cdots ,{\bar{q}}_{T_{1}}\right\}$ have to belong to $Q_{\mathrm{free}}$.

### 2.2. Collision Detection in Workspace Using the Oriented Bounding Box (OBB)

Even though the path planning algorithm mostly works in the configuration space Q, the path generated by the path planning algorithm has to make sure that the collision does not occur when the robot moves in workspace W. The workspace W is a space where an actual robot works, and is the subset of 3-dimensional Euclidean $R^{3}$. It is important to have a collision detection method to confirm ${\bar{q}}_{t}\in Q_{\mathrm{free}}$ in the middle of running the path planning algorithm [6,33]. To detect the collision in the workspace W, the oriented bounding boxes (OBB) [34] are employed for modeling all the 3D objects and finding the boundary in the 3D environment of the workspace including robots, obstacles, and ground. Simply speaking, any objects in the workspace are represented as boxes surrounding them in order to check collision.

Since objects in the workspace are modeled in the form of the OBB, the collision checking between two boxes has 15 cases: 3 faces of the first box, 3 faces of the second box, and 9 edge combinations between the first and second boxes [35]. By checking these 15 cases made by two OBBs, the collision detection between two boxes can be tested. Based on such a collision check for two boxes, the higher level collision checking between one robot and one obstacle can be implemented by repeated application of collision checking for two OBBs [36]. For the multi-arm manipulator case, each arm is considered as a moving obstacle to the others. Hence, again, collision checking in the workspace of the multi-arm manipulator can be tested using collision checking of OBBs for obstacles and robots.

### 2.3. Reinforcement Learning

Reinforcement learning is a decision making procedure for Markov decision process (MDP)  [37] which is defined by the tuple {S,A,P,r,γ} where *S* is the set of states, *A* the set of actions, *P* the transition probability, r(s,a) the reward function, and γ the discount factor [38]. The transition probability $P(s^{\prime }|s,a)$ is the probability that the current state s∈S moves to the next state $s^{\prime }$ when the action a∈A is applied to the environment at the current state s∈S. The policy of the agent denoted by π(a|s) is the distribution of action *a* for each state s∈S. At each time step *t*, the agent chooses action $a_{t}\in A$ based on the policy π:S→A. When the decided action is applied to the environment, the environment returns both the next state $s_{t+1}\in S$ and reward $r_{t+1}\in R$ from the transition probability P:S×A→P(A) and the reward function r:S×A→R. Conceptually, when the agent learns the optimal policy, the policy is determined such that it maximizes the expected return $E_{\pi }\left[\sum_{k=0}^{\infty }\gamma^{k}r_{t+k+1}\right]$. This learning procedure is repeated until the expected return converges. However, since it is not possible to compute the expected return in practice, its estimated value, called optimal value function, is maximized. With this framework, there are two approaches: value-based and policy-based approaches. In the value-based approach, the optimal value function is estimated. For instance, deep Q-network (DQN) approximates the optimal value function using deep neural networks. When the optimal value function is estimated, the corresponding optimal policy is derived from the approximated value function. On the other hand, the policy-based approach (also called the policy gradient) computes the optimal policy directly from the agent’s experience. Representatives of the policy gradient are REINFORCE, actor–critic method, deterministic policy gradient (DPG), deep DPG (DDPG), asynchronous advantage actor–critic (A3C), trust region policy optimization (TRPO), maximum a posteriori policy optimization (MPO) and distributed distributional DDPG (D4PG) [39,40,41,42,43]. In general, it is known that the policy gradient outperforms the value-based approaches, especially in the continuous action task [41,44].

In the reinforcement learning, the training performance depends heavily on sampled data consisting of the current state $s_{t}$, action $a_{t}$, next state $s_{t+1}$ and reward $r_{t+1}$. To enhance sample efficiency, the agent saves the samples in the memory first and use the saved samples taken from the memory. Replay memory [45] and HER [28] are tailored methods for this. In this paper, a state-of-the-art policy gradient method, known as soft actor–critic (SAC) [27] with HER, is employed for the multi-arm manipulator path planning algorithm.

## 3. Method

### 3.1. Path Planning for the Multi-Arm Manipulator and Augmented Configuration Space

For the path planning of a multi-arm manipulator, it is assumed that there are *m* identical robot manipulators and each robot has *n* joints. Let $q_{t}^{i}\in R^{n}$ denote the value of the joint angles of the *i*th manipulator at the *t*th iteration in the proposed deep reinforcement learning-based path planning algorithm, and $q_{t,j}^{i}\in R$ represents the *j*th element of $q_{t}^{i}\in R^{n}$, i.e., the value of the *j*th joint of the *i*th manipulator. Similarly to the path planning for a single robot manipulator, the path planning problem for the multi-arm manipulator is to find the shortest and collision-free path for a given starting configuration $q_{0}$ and goal configuration $q_{\mathrm{goal}}$ where

$$q_{0}=\left[\begin{array}{c} q_{\mathrm{init}}^{1}\\ q_{\mathrm{init}}^{2}\\ \vdots \\ q_{\mathrm{init}}^{m} \end{array}\right],\;q_{\mathrm{goal}\;}=\left[\begin{array}{c} q_{\mathrm{goal}}^{1}\\ q_{\mathrm{goal}}^{2}\\ \vdots \\ q_{\mathrm{goal}}^{m} \end{array}\right],$$

$q_{\mathrm{init}}^{i}$ and $q_{\mathrm{goal}}^{i}$ represent starting and goal configuration of the *i*th manipulator, respectively.

In order to design a path planning algorithm for the multi-arm manipulator, it is difficult to define $Q_{\mathrm{free}}$ and $Q_{\mathrm{collide}}$ for each arm if each arm is considered independently since the multi-arm collaborate (i.e., move together) in the same workspace and each arm is an obstacle to the others in the workspace. To cope with this situation, one remedy is to view the multi-arm manipulator as one virtual single-arm manipulator whose number of the joint is nm. Then, the virtual manipulator’s movement is described by augmenting the configuration of each arm. In other words, the state of the virtual manipulator is defined by

(1) $$q_{t}=\left[\begin{array}{c} q_{t}^{1}\\ q_{t}^{2}\\ \vdots \\ q_{t}^{m} \end{array}\right]\in R^{nm},\;\;q_{t}^{i}=\left[\begin{array}{c} q_{t,1}^{i}\\ q_{t,2}^{i}\\ \vdots \\ q_{t,n}^{i} \end{array}\right]\in R^{n},\;\;i\in \left\{1,2,\cdots ,m\right\}.$$

Consequently, the resulting augmented configuration space is defined by $Q^{a}:=Q_{\mathrm{free}}^{a}⋃Q_{\mathrm{collide}}^{a}$ where

$$Q_{\mathrm{free}}^{a}=\underset{m\;\mathrm{times}}{\underset{︸}{Q_{\mathrm{free}}\times \cdots \times Q_{\mathrm{free}}}}\subset R^{nm}\;\;\mathrm{and}\;\;Q_{\mathrm{collide}}^{a}=\underset{m\;\mathrm{times}}{\underset{︸}{Q_{\mathrm{collide}}\times \cdots \times Q_{\mathrm{collide}}}}\subset R^{nm},$$

$Q_{\mathrm{free}}^{a}$ denotes the collision-free space for $q_{t}$ and $Q_{\mathrm{collide}}^{a}$ is the corresponding collision space. Then, the path planning problem for the multi-arm manipulator can be redefined by the path planning problem for the one virtual single-arm manipulator with a given starting configuration $q_{0}\in Q_{\mathrm{free}}^{a}$ and goal configuration $q_{\mathrm{goal}}\in Q_{\mathrm{free}}^{a}$.

### 3.2. Multi-Arm Manipulator Markov Decision Process (MAMMDP)

**Figure 1:**
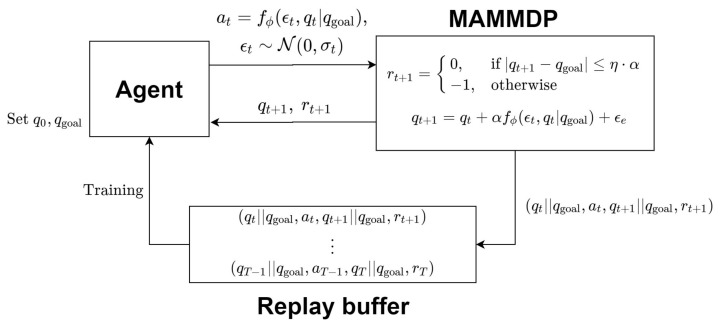
Multi-Arm Manipulator Markov Decision Process (MAMMDP).

For the sake of applying deep reinforcement learning to path planning algorithm design for multi-arm manipulators, a multi-arm manipulator MDP (MAMMDP), i.e., the tuple {S,A,P,r,γ}, is defined. The structure of MAMMDP can be seen in Figure 1. The current state of *m* manipulators $q_{t}\in Q_{\mathrm{free}}^{a}\subset R^{nm}$ is the *n* joint values of the *m* manipulators. The current action $a_{t}\in A$ is defined as a change of the state in the augmented configuration space $Q_{\mathrm{free}}^{a}$. The action is generated by $a_{t}=f\left(e_{t},q_{t}\right)$ where $e_{t}$ follows the Gaussian distribution $N(0,\sigma_{t})$ with $\sigma_{t}$ being the variance, and $f(e_{t},q_{t})$ generates a stochastic action using noise $e_{t}$ and state $q_{t}$. Actually, function f(·) is a sampler. In other words, the action is sampled from a probability distribution. Then, the next state is calculated as the sum of the current state and action such as ${\hat{q}}_{t+1}=q_{t}+\alpha a_{t}+\epsilon_{e}$ where tuning parameter α is the geometric mean of the maximum variations of each joint at a time and $\epsilon_{e}∼N\left(0,\sigma_{e}\right)$ and $\epsilon_{e}$ is an environment noise. When ${\hat{q}}_{t+1}\in Q_{\mathrm{collide}}^{a}$ occurs, the next state becomes the previous state, i.e., it stays at the current state.

Such an iteration of computing, implementing the action, and obtaining the state and reward are repeated until the next state reaches the goal point $q_{\mathrm{goal}}$, namely $q_{t+1}=q_{\mathrm{goal}}$, or stops when the number of the iteration hits *T* where *T* is the predefined maximum number of the iteration. However, in practice, $q_{t+1}=q_{\mathrm{goal}}$ rarely happens, at least, due to numerical problems. Hence, instead of that, a relaxed terminal condition $∥q_{t+1}-q_{\mathrm{goal}}∥\leq \eta \cdot \alpha$ is used where ∥·∥ denotes the norm of a vector and tuning parameter η meets 0<η<1. Note that the terminal condition means that the iteration stops when the next state reaches a small neighborhood of the goal configuration.

When the next state is not the goal state, the corresponding reward is −1 while the reward is 0 if the agent reaches the goal. Hence, at the end of the iteration, if the agent does not reach the goal, then the total reward is −T. On the other hand, if the iteration ends at a certain iteration $T_{1}<T$, then the total reward becomes $-(T_{1}-1)$. In summary, the state transition and reward function $r(q_{t},a_{t})$ are defined as follows.

(2) $$q_{t+1}=\left\{\begin{array}{cc} {\hat{q}}_{t+1}, & \mathrm{if}\;{\hat{q}}_{t+1}\in Q_{\mathrm{free}}^{a}\\ q_{t}, & \mathrm{if}\;{\hat{q}}_{t+1}\notin Q_{\mathrm{free}}^{a} \end{array}\right.,\;\;\;\;\;\;r_{t+1}=\left\{\begin{array}{cc} 0, & \mathrm{if}\;\;|q_{t+1}-q_{\mathrm{goal}}|\leq \eta \cdot \alpha \\ -1, & \mathrm{if}\;\;q_{t+1}\in Q_{\mathrm{collide}}^{a}\\ -1, & \mathrm{if}\;q_{t+1}\in Q_{\mathrm{free}}^{a} \end{array}\right.$$

The goal of reinforcement learning is to find the optimal policy maximizing the total reward that the agent gets in path planning iteration. If the iteration ends in a finite time $T_{1}<T$, the agent can learn to find a path to reach the goal by maximizing the total reward since the graph is connected. Besides, by trying to reduce the ending $T_{1}$ during training, the agent can seek an as short path as possible. However, when the agent is not trained sufficiently, it is hard to find a goal since the action is determined mainly by only the exploration method (e.g., random action sampling). This means that there are many iterations which fail to reach the goal until the agent is well trained. In this case, there are only few state, action, and reward trajectories which can contribute to learning (i.e., higher total reward). MDP with such a problem is called MDP with sparse reward and the path planning problem for the robot arm manipulator exactly falls into MDP with sparse reward. To overcome this problem in this paper, a path planning algorithm is designed based primarily on both recently proposed soft actor–critic (SAC) [27] and hindsight experience replay (HER) [28]. SAC is famous for its outstanding exploration performance and HER enhances the sample efficiency in deep reinforcement learning for MDP with sparse reward. The details of the proposed algorithm are presented in the next subsection.

### 3.3. Soft Actor-Critic (SAC)

MAMMDP has a continuous action, i.e., the agent’s action of MAMMDP is a continuous joint value and there are several policy gradient-based reinforcement learning methods for MDP with continuous action such as [27,41,46]. This section reviews briefly the main result of [27,47] and it is presented how to design a SAC-based path planning for MAMMDP. SAC is a maximum entropy reinforcement learning which maximizes not only the expected sum of rewards but also the entropy of the policy. Namely, SAC computes the optimal policy by maximizing the expected sum of entropy augmented reward defined by

(3) $$J\left(\pi \right)=\sum_{t=0}^{T}E_{q_{t},a_{t}}\left[r\left(q_{t},a_{t}\right)+\beta H\left(\pi \left(a_{t}|q_{t}\right)\right)\right],\;\;\;\;H\left(\pi \left(a_{t}|q_{t}\right)\right)=-\sum_{a_{t}}\pi \left(a_{t}|q_{t}\right)\log\pi \left(a_{t}|q_{t}\right),$$

where π is the policy which is updated to find the maximum total reward. β is the temperature parameter and it regulates the entropy term against the reward. $H(\pi \left(a_{t}|q_{t}\right))$ denotes entropy.

Usually, the distribution of the policy has small variance and its center is near a specific action that leads to the high return. On the other hand, the entropy in the objective of SAC makes the variance of the policy distribution increases. In the policy, the increased variance of the distribution means that the policy has more various actions that can be chosen. Accordingly, more explorations are carried out in this method. Because the multi-arm manipulator path planning is a multi-dimensional problem, enhancing the exploration plays a pivotal role in obtaining an efficient path planning algorithm. In SAC, the agent maximizes not only the expected return but also the entropy that leads to a better exploration. Because of this, the agent can acquire the optimal path of the multi-dimensional problem.

**Remark** **1.** *As the policy is described by probability distribution in the case of a stochastic policy, the distribution of the policy should match that of Q-value. Besides, such a stochastic policy is heavily affected by the exploration. Because SAC is a maximum entropy reinforcement learning framework, it not only enhances the exploration but also finds multiple optimal policies, which is possible due to the fact that the distribution functions of the Q-value and the policy are getting match as the training is going on by SAC. In the path planning problem, in general, there can be multiple solutions for the optimal path.*

**Figure 2:**
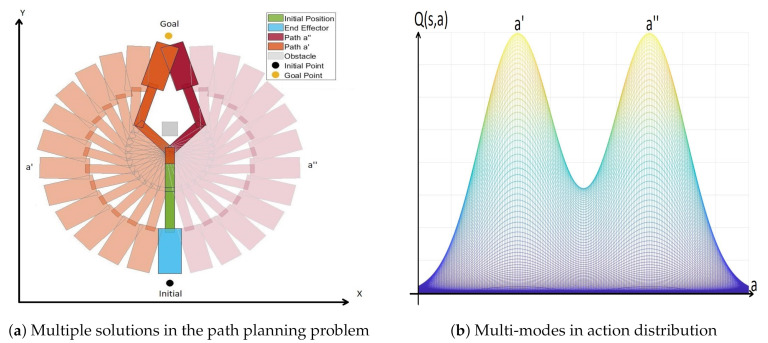
Multiple solutions in the path planning problem.

*For example, in Figure 2a, there are the initial point, goal point, and an obstacle in between them. In such a setting, it is obvious that there are two shortest paths between $q_{\mathrm{init}}$ and $q_{\mathrm{goal}}$. It is difficult for a deterministic policy to capture these two solutions. In contrast, the problem with multiple optimal solutions can be tackled by a stochastic policy with entropy augmented reward. Then, the probability distribution of the policy represents the information of two optimal solutions like in Figure 2b. Because of this, the stochastic policy such as SAC can find all optimal paths of a problem with multiple optimal solutions. This is why the SAC-based path planning can be effective for the path planning for a multi-arm manipulator which is highly dimensional.*

With the augmented reward function, the soft value function and soft Q-value function (or soft action value function) are defined respectively as follows:

(4) $$V\left(q_{t}\right)=E_{a_{t}∼\pi }\left[Q\left(q_{t},a_{t}\right)-\beta \log\pi \left(a_{t}|q_{t}\right)\right],$$

(5) $$Q\left(q_{t},a_{t}\right)=r\left(q_{t},a_{t}\right)+\gamma E_{q_{t+1}∼p}\left[V\left(q_{t+1}\right)\right].$$

These functions are evaluated at $q_{t}$ and $a_{t}$, and tell about how much reward can be obtained in the future. Soft Q-value $Q(q_{t},a_{t})$ is used to train the agent’s policy and the soft state value $V(q_{t})$ is needed to estimate the soft Q-value. In SAC, the policy, the soft state value, and the soft Q-value functions are approximated by deep neural networks (DNNs). Each of them is parameterized by ϕ,ψ, and θ, respectively. Besides, there exists another target network with parameter $\bar{\psi }$. The target network is used to improve learning performance for the soft Q-value and makes the learning more stable. In addition, the technique using the double Q-value functions is employed [27,46,48]. Consequently, there are five DNNs in SAC: the parameterized policy $\pi_{\phi }\left(a_{t}|q_{t}\right)$, the soft state value $V_{\psi }\left(q_{t}\right)$ with target $V_{\bar{\psi }}\left(q_{t}\right)$ and the two soft Q-values $Q_{\theta_{1,2}}\left(q_{t},a_{t}\right)$. Finally, the action is sampled from $f_{\phi }\left(e_{t},s_{t}\right)$, i.e., $a_{t}=f_{\phi }\left(e_{t},s_{t}\right)$.

To find the optimal policy, soft-Q value and soft state value, the stochastic gradient descent method is applied to their objective functions. The DNN for the soft state value is trained to minimize the mean squared error with estimated soft state value given by

(6) $$J_{V}\left(\psi \right)=E_{q_{t}}\left[\frac{1}{2}{\left(V_{\psi }\left(q_{t}\right)-E_{a_{t}}\left[\min_{k=1,2}Q_{\theta_{k}}\left(q_{t},a_{t}\right)-\beta \log\pi_{\phi }\left(a_{t}|q_{t}\right)\right]\right)}^{2}\right].$$

In this function, the minimum of the soft Q-value is taken over two Q-value functions parameterized by $\theta_{1}$ and $\theta_{2}$. This helps to avoid overestimation that gives high ratings to inappropriate Q-values [46]. The soft Q-value is trained to predict how much return can be obtained from pair ($q_{t},a_{t}$) and this is also done by minimizing the Bellman equation given by

(7) $$J_{Q}\left(\theta_{k=1,2}\right)=E_{q_{t},a_{t}}\left[\frac{1}{2}{\left(Q_{\theta_{k=1,2}}\left(q_{t},a_{t}\right)-\left(r\left(q_{t},a_{t}\right)+V_{\bar{\psi }}\left(q_{t+1}\right)\right)\right)}^{2}\right].$$

Both $\theta_{1}$ and $\theta_{2}$ are trained with their Q-values. The minimum of these Q-values is also used for the policy network learning. The objective function of the policy network is the information projection between the distribution of the current policy and the distribution of the minimum Q-value as follows.

(8) $$J_{\pi }\left(\phi \right)=E_{q_{t},a_{t}}\left[\log\pi_{\phi }\left(a_{t}|q_{t}\right)-\min_{k=1,2}Q_{\theta_{k}}\left(q_{t},a_{t}\right)\right].$$

As a matter of fact, this objective function is a simplified Kullback-Leibler (KL) divergence between the policy and Q-value [27]. After minimizing this function with respect to parameter ϕ, the distribution of the policy becomes proportional to the distribution of Q-value. At each training step, after training the other parameters, the parameter of the target soft state value $\bar{\psi }$ is updated according to $\bar{\psi }\leftarrow \tau \psi +\left(1-\tau \right)\bar{\psi }$ by a tuning parameter τ∈[0,1]. Since SAC is based on off-policy actor–critic  [27,44], we do not need to use the current policy for training the networks. Instead, the transition tuples $\left(q_{t},a_{t},q_{t+1},r\left(q_{t},a_{t}\right)\right)$ are collected in every iteration and then, stored in experience replay memory D [45]. At the beginning of the training procedure, a bundle of these tuples is sampled and they are used to compute the expectation in the objective functions.

### 3.4. Hindsight Experience Replay (HER)

In the MAMMDP under consideration, the agent does not receive negative rewards only when the goal is reached. As mentioned before, since the MAMMDP is an MDP with sparse reward, the agent suffers from low sample efficiency. To improve the sample efficiency, the hindsight experience replay (HER) [28] method is used to deal with the measured samples. Suppose that the iteration in the algorithm ends without reaching the goal state, and let the corresponding trajectory becomes a failed episode $e=[\left(q_{0},a_{0}\right),\left(q_{1},r_{1},a_{1},\cdots ,\left(q_{T},r_{T}\right)\right)]$. Since the agent does not arrive at the goal state in this episode, it follows that $q_{T}\neq q_{\mathrm{goal}}$, which means that the agent does not learn much from this episode. To resolve this problem, HER defines a new goal state $q_{\mathrm{goal}}^{\prime }$, chooses one state in the failed episode, let say $q_{t^{\prime }},\;t^{\prime }\in \left\{1,2,\cdots ,T\right\}$, and sets $q_{goal}^{\prime }=q_{t^{\prime }}$. Then, the modified episode $e^{\prime }=\left[\left(q_{0},a_{0}\right),\left(q_{1},r_{1},a_{1},\cdots ,\left(q_{t^{\prime }},r_{t^{\prime }}^{\prime }\right)\right)\right]$ becomes a successful episode since $q_{t^{\prime }}=q_{\mathrm{goal}}^{\prime }$ is achieved where $r_{t^{\prime }}^{\prime }\equiv 0$. Then, this modified episode can improve the learning performance and this is how HER enhances the sample efficiency.

**Figure 3:**
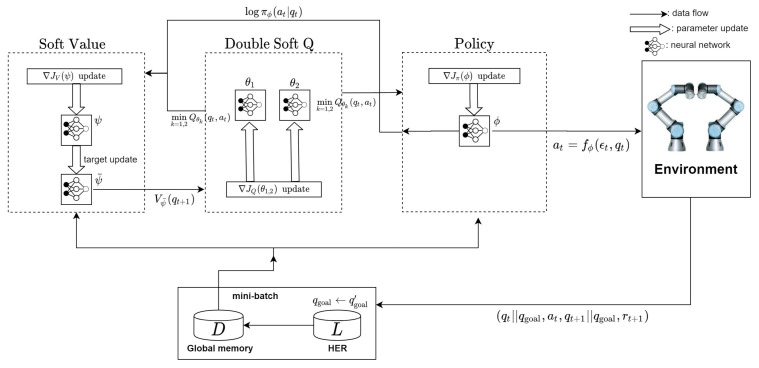
Architecture of the proposed SAC-based path planning algorithm.

**Figure 4:**
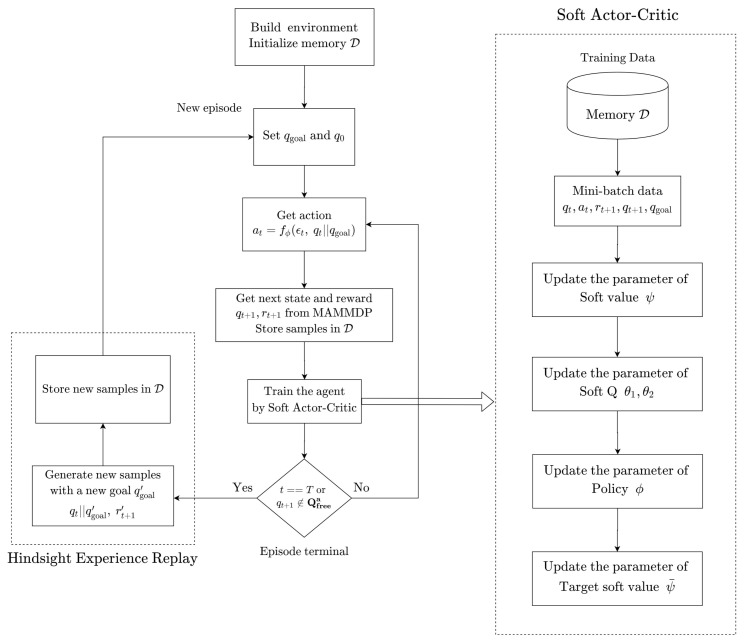
Flow chart of the proposed SAC-based path planning algorithm.

The proposed SAC-based path planning algorithm is depicted in Figure 3 and Figure 4. Also, the details of the implementation of the proposed method can be found in Appendix A.

## 4. Results and Discussion

In this section, the proposed SAC-based path planning algorithm is applied to two real 3-DOF open manipulators. The parameters of the 3-DOF manipulator are given in Table 1. To see the details of the 3-DOF open manipulator, see http://en.robotis.com/model/page.php?co_id=prd_openmanipulator.

The parameters used in the SAC-based path planning are described in Table 2.

**Figure 5:**
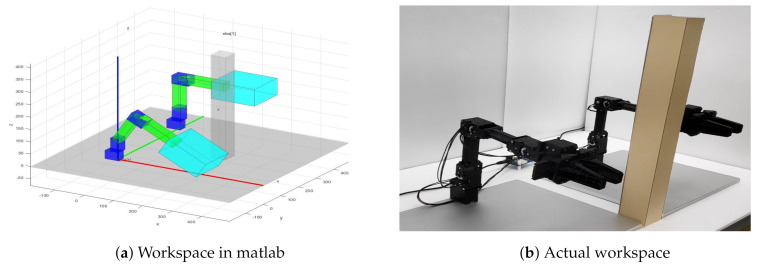
The workspace of robots.

For the experiment, the workspace in Figure 5 is considered. The size of the workspace is 90 cm × 60 cm × 47 cm. The bar in between two manipulators is an obstacle. Figure 5a represents the visualization of the workspace in Matlab and Figure 5b shows the actual workspace. The problem to be solved is to devise a path planning algorithm using the proposed method for each arm in this setting such that the shortest paths for each arm are computed when arbitrary start and goal positions for the end-effect of each arm are given. Note that the path planning for this problem setup is nontrivial since there is a common workspace in workspaces of each arm in addition to the obstacle.

**Figure 6:**
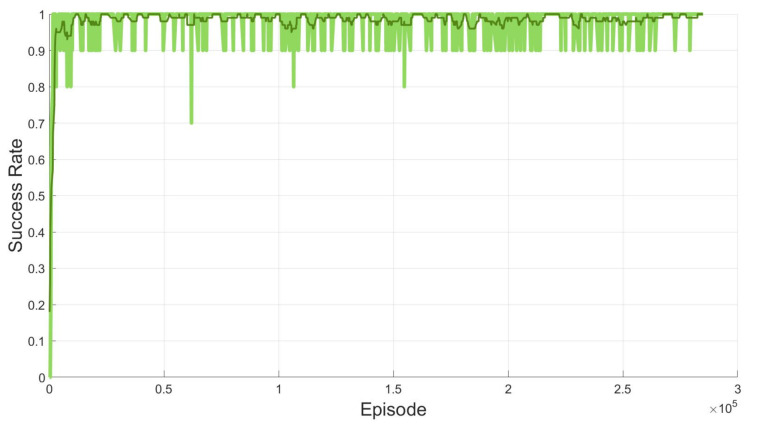
Success ratio by the proposed SAC-based path planning for two open manipulators.

In the training process, 300,000 episodes are implemented to train the networks in the proposed SAC-based path planning algorithm. In each episode, arbitrary start and goal locations belonging to the workspace are given and the agent is trained to find the shortest path between them by the proposed algorithm. Figure 6 shows that success ratio of the 300,000 episodes.

The success means that the path planning algorithm finds the shortest path successfully. In Figure 6, the green line denotes the moving average of success ratio of every 10 episodes where the success ratio is set to 1 when the shortest path is computed successfully, and 0 otherwise. The thick line is the moving average of the green lines. As seen in Figure 6, the proposed path planning algorithm finds the path without fail in most cases.

**Figure 7:**
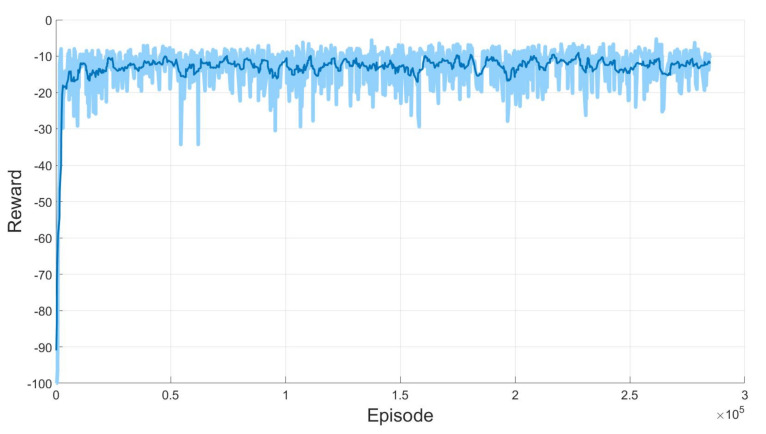
Reward from learning for two 3-DOF manipulators.

In addition to the success ratio, the reward is also an important aspect to evaluate the training performance of any reinforcement learning-based algorithm. In the proposed algorithm, the reward value can be interpreted as the optimal time generating the path due to the definition of the reward Function (2). Figure 7 depicts the reward for the 300,000 episodes.

In Figure 7, the light blue line denotes the actual reward and the thick blue line describes the moving average of the light blue line. In view of Figure 7, it can be seen that the reward converges very quickly as the episode increases, which implies the algorithm finds the shortest path efficiently and consistently. Figure 6 and Figure 7 demonstrate that the agent is trained well by the proposed SAC-based path planning algorithm such that it not only reaches the given goal mostly but also maximizes the return due to the quickly converged reward, which means that the training process is done successfully.

**Figure 8:**
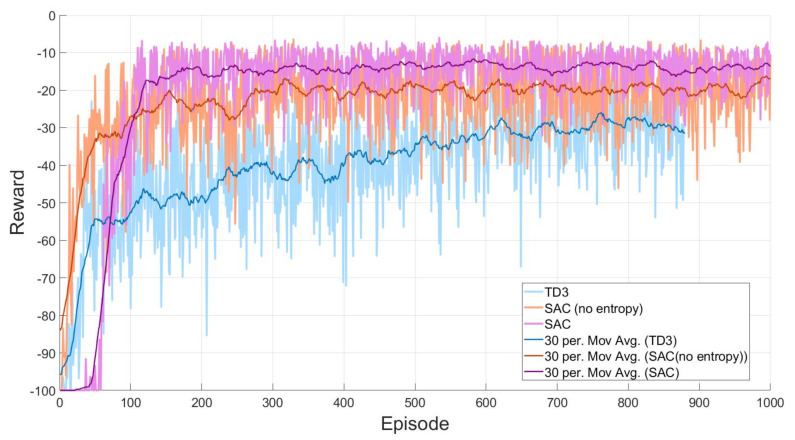
Comparison of the training performances between TD3 and SAC.

To see the advantage of the proposed method more, we implement other algorithms in the training process and plot the data in one graph for comparison. Figure 8 shows the training results made by three different path planning algorithms based on deep reinforcement learning. The light blue line is the actual reward from TD3 (Twin Delayed Deep Deterministic Policy Gradient) and the thick blue line is the moving average of the light blue line. Then, the orange line indicates SAC without entropy (β=0) and the purple line is the original SAC with entropy. For SAC without entropy, the moving average is in the thick orange line and the moving average of SAC is in the thick purple line. Basically, because β is equal to zero in SAC without entropy, the objective function of SAC without entropy is exactly the same as the objective function of TD3. At the beginning of the training, we can see that SAC has a late transient response than TD3 and SAC without entropy. This is because SAC has an entropy factor in the objective function. Moreover, the TD3 and SAC without entropy have the same objective function that only maximizes the expected reward without the entropy. However, at the steady-state, SAC can reach a higher reward value than the other algorithms. This means that the trained actor from SAC can find shorter path than the other algorithms. This is because SAC aims to maximize not only the expected reward but also the entropy.

Once the actor is trained well, the actor-network can be used to compute the shortest path for a given arbitrary start and goal location in the workspace. As the output of the trained actor is only the mean of the stochastic policy, the output does not have any noise from the sampling distribution. To be specific, in testing step, ($q_{\mathrm{init}},q_{\mathrm{goal}}$) is injected into the trained actor in the beginning. Then, its output is the mean action (without noise) which is applied to the environment. The measurement, including the environment noise, is the next state which is injected into the actor-network together with $q_{\mathrm{goal}}$. Then, the actor-network generates the action again. This procedure is repeated until the goal state is reached. In other words, the trained actor generates the following path to the goal state.

$$\underset{\mathrm{Input to the actor}}{\underset{︸}{\left(q_{\mathrm{init}},q_{\mathrm{goal}}\right)}}\;\to \;\underset{\mathrm{Output of the actor}}{\underset{︸}{a_{0}}}\;\to \;\underset{\mathrm{Measurement}}{\underset{︸}{q_{1}}}\;\to \;\underset{\mathrm{Input to the actor}}{\underset{︸}{\left(q_{1},q_{\mathrm{goal}}\right)}}\;\to \;a_{1}\;\to \;q_{2}\;\cdots \;\left(q_{t},q_{\mathrm{goal}}\right)\;\to \;\cdots \;\to \;\left(q_{\mathrm{goal}},q_{\mathrm{goal}}\right).$$

**Figure 9:**
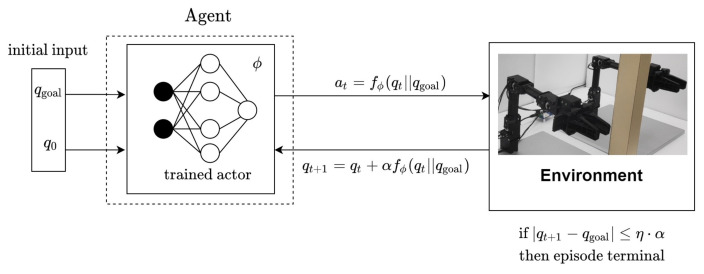
Path generation using the trained actor DNN.

Figure 9 depicts the path inference procedure.

**Figure 10:**
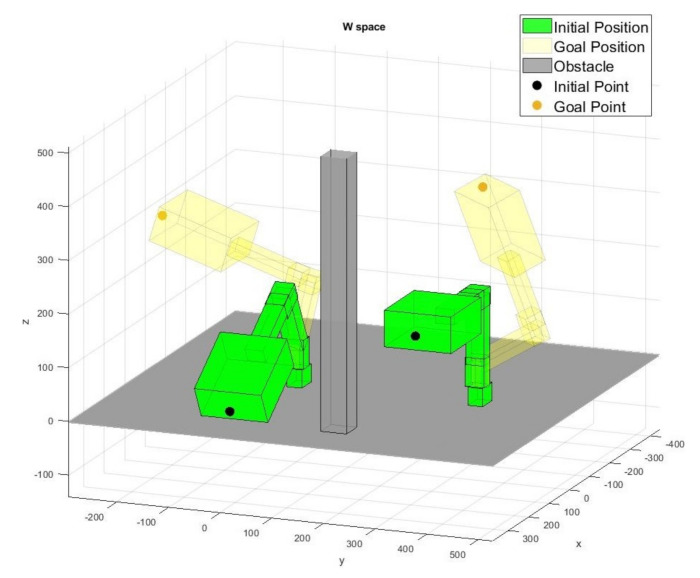
Initial and goal positions in the workspace.

Figure 10 shows an example of the start position $q_{\mathrm{init}}$ and goal position $q_{\mathrm{goal}}$ in the workspace where the green robot position is the initial position and the light yellow denotes the goal position. As explained before, if $q_{\mathrm{init}}$ and $q_{\mathrm{goal}}$ are given to the trained agent, the shortest path is computed.

**Figure 11:**
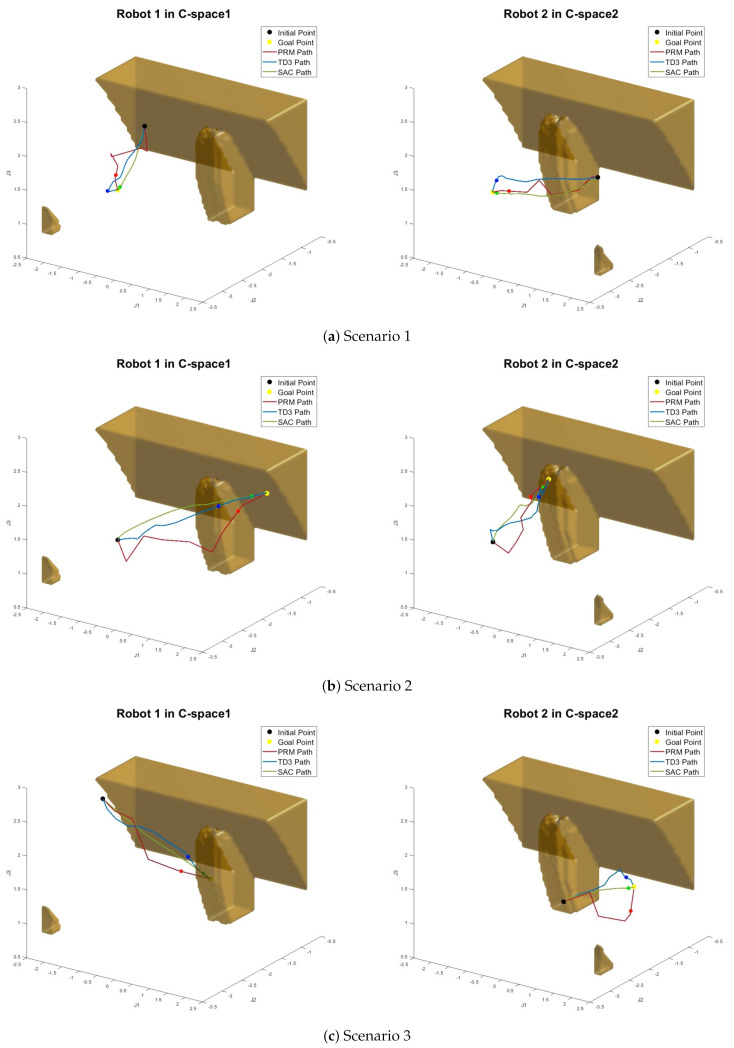
Path generation by SAC with HER for arbitrary initial and goal points in each C-space robot.

Figure 11 depicts the configuration space ($\subset R^{3}$) of each arms extracted from the augmented configuration space ($\subset R^{6}$). The left figure in Figure 11 shows the configuration space of the first arm and the right figure that of the second arm. Figure 11 shows several examples of resulting paths computed by the actor for given arbitrary start and goal locations in the configuration space. The two configuration spaces of each robot are used for visualizing the generated path. The lines in Figure 11 represent the joint movement of robots. In the configuration space, there are two kinds of collision space: the static collision space as a static obstacle and dynamic collision space as a moving obstacle (the other robot). In Figure 11, the static obstacle is represented by the brown area of the configuration space. The video clips for the simulation and experiment can be found at https://sites.google.com/site/cdslweb/publication/sacpath.

For comparison, the other method like PRM (35,000 sampled point graph) and TD3 with HER also generate a path with the same task. In Figure 11, the red lines denote the result by PRM method and the blue lines by TD3 with HER, and the green lines by the proposed method. The yellow points describe the goal position of each robot and the black points tell the start position of each robot. As shown in Figure 11, the proposed path planning using SAC with HER method leads to smoother and shorter paths than the other algorithms.

**Figure 12:**
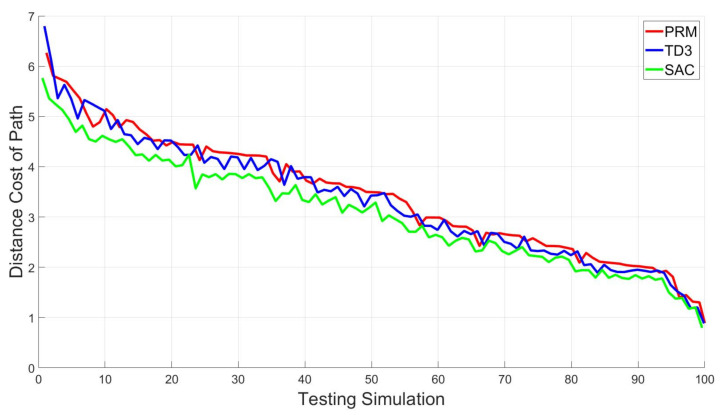
Comparison of paths by PRM, TD3 with HER and the proposed method.

To see the quality of the proposed method, 100 simulations of the arbitrary initial and goal points are implemented using the PRM, TD3 with HER, and SAC with HER simultaneously. Since the result paths can be interpreted as performance, the 100 generated paths are used for comparison in Figure 12. As seen in Figure 12, the proposed path planning using SAC with HER always finds the shortest path for all simulation cases. For the result shown in Figure 12, it takes 0.1385 s on average computing the shortest path for a start point and goal point. This means that the proposed SAC-based path planning can generate the optimal path quickly so that it works for the multi-arm manipulator in real-time such as industrial application. Note that the training is done off-line.

**Figure 13:**
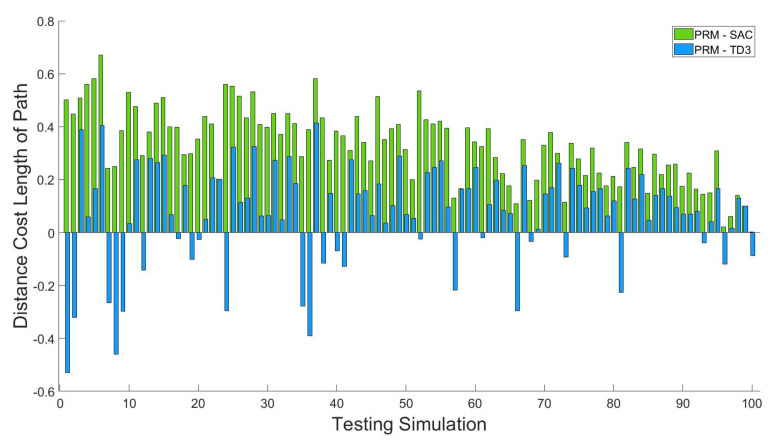
Path difference between PRM-TD3 with HER and PRM-SAC with HER by PRM.

For better comparison, the paths are calculated for 100 simulations using different algorithms and are drawn in Figure 13.

From Figure 13, we can see that SAC- or TD3-based path planning is better than PRM, and SAC-based algorithm is better than TD3-based algorithm. In Table 3, three path planning methods are compared. The average path cost is the average of the path length from testing simulation in Figure 12, and the cost percentage is another ratio of the path cost where PRM’s average path cost is defined as 100%. As shown in Table 3, SAC leads to the lowest cost compared with others. The roughness is a metric representing how smooth the path is [49]. It is evaluated by the mean squared error with second derivative of the path. In other words, the roughness is defined by $l_{\mathrm{roughness}}\left(q_{1},q_{2},\cdots ,q_{T}\right)=\frac{1}{T}\sum_{t=1}^{T}{\left(\frac{d^{2}q_{t}}{dt^{2}}\right)}^{2}$. As shown in Figure 11 and Table 3, the generated path by SAC is smoothest among the  others.

Such good performance of the proposed SAC-based algorithm is due to its better exploration performance coming from maximizing the entropy. In the maximum entropy framework, the agent explores the environment to maximize the return and the entropy. Because of this reason, the algorithm can find the optimal path in any task. However, in deterministic policy like TD3, the agent only maximizes the expected reward, which can lead to poor exploration performance.

In view of all simulation and experimental results, it is confirmed that the proposed method generates a shorter and smoother path than other algorithms. To be specific, notice that the optimal path of the proposed method is shorter by 9.66% than that of PRM on average. Compared with the TD3 with HER, on average, the paths by the proposed method are 7.6% shorter than those by TD3 with HER.

## 5. Conclusions and Future Work

This paper presents a deep reinforcement learning-based path planning algorithm for the multi-arm manipulator. In order to solve the high-dimensional path-planning problem, SAC (Soft Actor-Critic)-based algorithm is proposed. To deal with the multi-arm efficiently in configuration space, configuration spaces of each arm are augmented, and HER (Hindsight Experience Replay) is employed to enhance sample efficiency. Both the simulation and experiment show that the proposed algorithm finds the shortest path for arbitrary start and goal positions and the generated paths are shorter and smoother than those generated by existing results. Such results are made due to better exploration performance yielded by the entropy term in the objective function of SAC. Since the agent is trained off-line, the trained agent can generate the shortest path for an arbitrary start and goal positions quickly which means that the proposed algorithm can be used in real-time application.

This paper assumed that the work environment is static. Namely, the obstacles in the workspace do not change during the robot arm manipulator operation. Hence, a natural future work is the path planning for dynamic environment. For instance, if obstacles are moving in workspace, it is nontrivial to define Markov decision process in order to design a path planning algorithm using reinforcement learning.

## Figures and Tables

**Table 1:** Parameters of the 3-DOF manipulator.

Name	Value	Notation
The number of joints for each manipulator	3	*n*
The number of manipulators	2	*m*
Dimension of $Q_{\mathrm{free}}^{a}$	6	n·m
Joint maximum	(140, −45, 150, 140, −45, 150)	
Joint minimum	(−140, −180, 45, −140, −180, 45)	

**Table 2:** Tuning parameters for the designed SAC with HER.

Name	Value	Notation
Policy network size	12∗800∗500∗400∗400∗300∗6	ϕ
Soft Q network size	18∗800∗500∗400∗400∗300∗1	$\theta_{1,2}$
Soft value network size	12∗800∗500∗400∗400∗300∗1	ψ
Learning rate	0.0001	
Replay memory size	$10^{6}$	D
Episode maximum step	100	*T*
Soft value target copy rate	0.005	τ
Mini batch size	512	*m*
Environment noise deviation	0.002	$\epsilon_{e}$
Action step size	0.3813	α
Goal boundary	0.2	η
Dicount factor	0.98	γ
Entropy temperature parameter	0.2	β

**Table 3:** Comparison of the proposed result with existing methods.

Method	Average Path Cost	Cost Percentage	Roughness
PRM	3.4162	100%	0.4153
TD3	3.3402	97.8%	0.0437
SAC	3.0862	90.3%	0.0206

**Figure A1:**
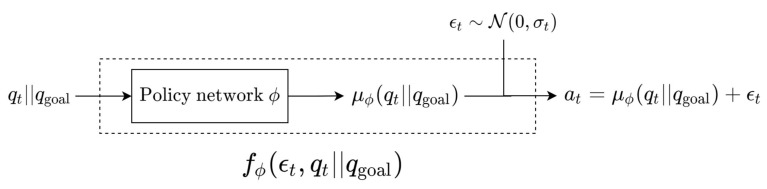
Reparameterized sampler for the continuous action sampling.

## Abbreviations

The following abbreviations are used in this manuscript:

MAMMDP	Multi-Arm Manipulator Markov Decision Process
SAC	Soft Actor-Critic
HER	Hindsight Experience Replay
AI	Artificial Intelligence
FMMs	Fast Marching Methods
PRM	Probabilistic Road Map
RRT	Rapid exploring Random Trees
DNN	Deep Neural Network
TD3	Twin Delayed Deep Deterministic Policy Gradient
MDP	Markov Decision Process
DOF	Degree of Freedom
OBB	Oriented Bounding Boxes
DQN	Deep Q-Network
DPG	Deterministic Policy Gradient
DDPG	Deep Deterministic Policy Gradient
A3C	Asynchronous Advantage Actor-Critic
TRPO	Trust Region Policy Optimization
MPO	Maximum a Posteriori Policy Optimisation
D4PG	Distributed Distributional Deep Deterministic Policy Gradient
KL	Kullback-Leibler

## Appendix A

In this section, the proposed algorithm is explained in terms of implementation. The proposed reinforcement learning-based path planning algorithm starts from goal-conditioned reinforcement learning. In this setting, the policy of the agent is defined like $\pi (a_{t}|\;q_{t}||q_{\mathrm{goal}})$ which is probability of the action $a_{t}$ where current state $q_{t}$ and goal state $q_{\mathrm{goal}}$ are given. $q_{t}\left|\right|q_{\mathrm{goal}}$ is the concatenated vector of the joint values and is the input of the policy π(·,·) where || denotes the concatenation. This setting is the same as that of the original HER (Hindsight Experience Replay) algorithm [28]. Also, the soft Q-value function and the soft state-value function depend not only on a state-action pair but also on a goal where the soft Q-value function is $Q(q_{t}||q_{\mathrm{goal}},a_{t})$ and the soft state-value function is $V(q_{t}||q_{\mathrm{goal}})$.

In the HER, the additional goal $q_{\mathrm{goal}}^{\prime }\in \left\{q_{1},q_{2},\cdots ,q_{T}\right\}$ is selected to generate a new sample $q_{t}\left|\right|q_{\mathrm{goal}}^{\prime }$ at the end of every episode. The additional goal is randomly chosen from $\left\{q_{1},q_{2},\cdots ,q_{T}\right\}\subset Q_{free}^{a}$ and $q_{\mathrm{goal}}^{\prime }\notin Q_{collide}^{a}$. When the agent is trained with this new sample, the sample efficiency of training process can be improved. Since the reward function of MAMMDP is based on a sparse reward setting and has no information on the direction or distance to the goal state, it is extremely difficult to train the agent without HER algorithm. This gets worse as the dimension of the path planning problem increases. By using this technique, we can increase the number of additional goals at each episode to speed up the training. However, when we compare the performance between TD3 and SAC in Figure 8, only one additional goal is used in every episode.

By utilizing reparameterized sampler $f_{\phi }(\epsilon_{t},q_{t}\left|\right|q_{\mathrm{goal}})$ and without using original stochastic policy $\pi_{\phi }(a_{t}|\;q_{t}\left|\right|q_{\mathrm{goal}})$, SAC produces a continuous action as its output [27]. Then, for the sampling noise, $\epsilon_{t}$ is given from the Gaussian distribution where $\epsilon_{t}∼N\left(0,\sigma_{t}\right)$ and $\sigma_{t}$ is a variance. To be specific, the outputs of the policy network are mean $\mu_{\phi }$ and variance $\sigma_{t}$. According to the objective Functions (6) and (8), we need to calculate $E_{a_{t}}\log\pi_{\phi }(a_{t}|\;q_{t}\left|\right|q_{\mathrm{goal}})$ where $a_{t}=f_{\phi }(\epsilon_{t},q_{t}\left|\right|q_{\mathrm{goal}})$. This is nothing but log-likelihood with the probability of action $\pi_{\phi }(a_{t}|\;q_{t}\left|\right|q_{\mathrm{goal}})$. Since $a_{t}$ is sampled from Gaussian distribution, this log-likelihood is given by

(A1) $$E_{a_{t}}\log\pi_{\phi }(a_{t}|\;q_{t}\left|\right|q_{\mathrm{goal}})=-\frac{l}{2}\log\left(2\pi \right)-\frac{l}{2}\log\left(\sigma_{t}^{2}\right)-\frac{1}{2\sigma_{t}^{2}}\sum_{i=1}^{l}(a_{i}-\mu_{\phi }(q_{t}\left|\right|q_{\mathrm{goal}})),$$

where *l* is the number of samples. Variance $\sigma_{t}$ can be viewed as a design parameter or can be tunable via learning. In this paper, it is fixed as a constant. Figure A1 describes how the action is sampled.

Soft Actor-Critic, unlike the original policy gradient algorithm, maximizes the expected sum of entropy augmented reward in the Equation (3). The entropy in the reward function can be evaluated by the Equation (A1) and this entropy augmented reward Function can be seen in the objective Functions (6)–(8). The order of optimizing objective Functions (6) and (7) is not important, but the optimization of objective Function (8) must be done after optimization of objective Functions (6) and (7). That is because the policy has to be updated towards the distribution of new soft Q-function. Moreover, these three optimizations are done by stochastic gradient descent with Adam optimizer and fixed learning rates. The gradients of the objective Functions (6)–(8) are defined as follows:

(A2) $$\begin{array}{cc} \;\;\;\;\;\nabla_{\psi }J_{V}\left(\psi \right) & \;\;=\;\;E_{q_{t}}[\frac{1}{2}(\nabla_{\psi }V_{\psi }(q_{t}\left|\right|q_{\mathrm{goal}})\;-\;E_{a_{t}}[\min_{k=1,2}Q_{\theta_{k}}(q_{t}\left|\right|q_{\mathrm{goal}},a_{t})\;-\;\beta \log\pi_{\phi }(a_{t}|\;q_{t}\left|\right|q_{\mathrm{goal}}{)])}^{2}], \end{array}$$

(A3) $$\begin{array}{cc} \nabla_{\theta_{k=1,2}}J_{Q}\left(\theta_{k=1,2}\right) & =E_{q_{t},a_{t}}[\frac{1}{2}(\nabla_{\theta_{k=1,2}}Q_{\theta_{k=1,2}}(q_{t}\left|\right|q_{\mathrm{goal}},a_{t})-(r_{t+1}+V_{\bar{\psi }}(q_{t+1}\left|\right|q_{\mathrm{goal}}{)))}^{2}], \end{array}$$

(A4) $$\begin{array}{cc} \nabla_{\phi }J_{\pi }\left(\phi \right) & =E_{q_{t},a_{t}}[\beta \nabla_{\phi }\log\pi_{\phi }(a_{t}|q_{t}\left|\right|q_{\mathrm{goal}})-\nabla_{\phi }\min_{k=1,2}Q_{\theta_{k}}(q_{t}\left|\right|q_{\mathrm{goal}},a_{t})] \end{array}$$

(A5) $$\begin{array}{cc} \; & \approx \beta \nabla_{\phi }\log\pi_{\phi }(a_{t}|q_{t}\left|\right|q_{\mathrm{goal}})+(\beta \nabla_{a_{t}}log\pi_{\phi }(a_{t}|q_{t}\left|\right|q_{\mathrm{goal}})\\ \; & \;\;\;\;\;\;\;\;\;\;-\nabla_{a_{t}}\min_{k=1,2}Q_{\theta_{k}}(q_{t}\left|\right|q_{\mathrm{goal}},a_{t}))\nabla_{\phi }f_{\phi }(\epsilon_{t},q_{t}\left|\right|q_{\mathrm{goal}}). \end{array}$$

Note that Equation (A5) is an approximated and decomposed form of Equation (A4) where $a_{t}=f_{\phi }(\epsilon_{t},q_{t}\left|\right|q_{\mathrm{goal}})$. These three gradients are used to solve each of the minimization problems. The complete algorithm is described in Algorithm 1.

**Algorithm 1:** Proposed SAC-based path planning algorithm for multi-arm manipulator.
1: Define MAMMDP and the augmented state $q_{t}$ and the state and goal state $q_{\mathrm{init}}$ and $q_{\mathrm{goal}}$ 2: Initialize network parameters $\psi ,\theta_{1,2},\phi$ 3: Initialize the parameter values of the target network $\bar{\psi }\leftarrow \psi$ 4: Initialize global replay memory D 6: **for**e=1 to *M***do** 7: Initialize local buffer L ▹ Memory for an episode 8: **for** t=0 to T−1 **do** 9: Randomly choose the goal and initial positions $q_{\mathrm{goal}},q_{\mathrm{init}}\in Q_{\mathrm{free}}^{a}$ 10: $a_{t}=f_{\phi }(\epsilon_{t},q_{t}\left|\right|q_{\mathrm{goal}}),\;\epsilon_{t}∼N\left(0,\sigma_{t}\right)$ 11: ${\hat{q}}_{t+1}=q_{t}+\alpha \cdot a_{t}+\epsilon_{e},\;\;\epsilon_{e}∼N\left(0,\sigma_{e}\right)$ 13: **if** ${\hat{q}}_{t+1}\in Q_{\mathrm{free}}^{a}$**then** ▹ Get next state and reward 14: $q_{t+1}\leftarrow {\hat{q}}_{t+1}$ 15: $r_{t+1}=-1$ 16: **else if** ${\hat{q}}_{t+1}\in Q_{\mathrm{collide}}^{a}$ **then** 17: $q_{t+1}\leftarrow q_{t}$ 18: $r_{t+1}=-1$ 19: **else if** $|q_{t+1}-q_{\mathrm{goal}}|\leq \eta \cdot \alpha$ **then** 20: $r_{t+1}=0$ 21: Terminate due to goal arrival 22: **end if** 24: Store the transition $\left(q_{t}||q_{\mathrm{goal}},a_{t},r_{t+1},q_{t+1}||q_{\mathrm{goal}}\right)$ in D,L 25: ▹ Parameters update 26: Sample mini-batch of *m* transitions $\left(q_{l}||q_{\mathrm{goal}},a_{l},r_{l+1},q_{l+1}||q_{\mathrm{goal}}\right)$ from D 27: $\;\;\;\;\;J_{V}\left(\psi \right)=E_{q_{l}}[\frac{1}{2}(V_{\psi }(q_{l}\left|\right|q_{\mathrm{goal}})-E_{a_{l}}[\min_{k=1,2}Q_{\theta_{k}}(q_{l}\left|\right|q_{\mathrm{goal}},a_{l})-\beta \log\pi_{\phi }(a_{l}|\;q_{l}\left|\right|q_{\mathrm{goal}}{)])}^{2}]$ 28: $\;\;\;\;\;J_{Q}\left(\theta_{k=1,2}\right)=E_{q_{l},a_{l}}[\frac{1}{2}(Q_{\theta_{k=1,2}}(q_{l}\left|\right|q_{\mathrm{goal}},a_{l})-(r_{l+1}+V_{\bar{\psi }}(q_{l+1}\left|\right|q_{\mathrm{goal}}{)))}^{2}]$ 29: $\;\;\;\;\;J_{\pi }\left(\phi \right)=E_{q_{l},a_{l}}[\beta \log\pi_{\phi }(a_{l}|q_{l}\left|\right|q_{\mathrm{goal}})-\min_{k=1,2}Q_{\theta_{k}}(q_{l}\left|\right|q_{\mathrm{goal}},a_{l})]$ 31: Each network parameters $\psi ,\theta_{1,2},\phi$ are updated by gradient descent 32: using $\nabla_{\psi }J_{V}\left(\psi \right),\nabla_{\theta_{1}}J_{Q}\left(\theta_{1}\right),\nabla_{\theta_{2}}J_{Q}\left(\theta_{2}\right),\nabla_{\phi }J_{\pi }\left(\phi \right)$ 34: Update state value target $\bar{\psi }\leftarrow \tau \psi +\left(1-\tau \right)\bar{\psi }$ 35: **end for** 37: **if** $q_{T}\neq q_{\mathrm{goal}}$**then** ▹ HER 38: Set additional goal $q_{\mathrm{goal}}^{\prime }\in \left\{q_{1},q_{2},\cdots ,q_{T}\right\}$ 39: **for** t=0 to T−1 **do** 40: Sample a transition $\left(q_{t}||q_{\mathrm{goal}},a_{t},r_{t},q_{t+1}||q_{\mathrm{goal}}\right)$ from L 41: **if** $|q_{t+1}-q_{\mathrm{goal}}^{\prime }|\leq \eta \cdot \alpha$ **then** 42: $r_{t+1}^{\prime }=0$ 43: **else** $r_{t+1}^{\prime }=-1$ 44: **end if** 45: Store the transition $\left(q_{t}||q_{\mathrm{goal}}^{\prime },a_{t},r_{t+1}^{\prime },q_{t+1}||q_{\mathrm{goal}}^{\prime }\right)$ in D 46: **end for** 47: **end if** 48: **end for**

## References

[1] Spong M., Hutchinson S., Vidyasagar M. (2006). Robot Modeling and Control.

[2] Albu-Schäffer A., Hirzinger G. (2001). A globally stable state feedback controller for flexible joint robots. Adv. Robot..

[3] Basile F., Caccavale F., Chiacchio P., Coppola J., Curatella C. (2012). Task-oriented motion planning for multi-arm robotic systems. Robot. -Comput.-Integr. Manuf..

[4] Shome R., Bekris K.E. Anytime Multi-arm Task and Motion Planning for Pick-and-Place of Individual Objects via Handoffs. Proceedings of the 2019 International Symposium on Multi-Robot and Multi-Agent Systems (MRS).

[5] Lynch K.M., Park F.C. (2017). Modern Robotics: Mechanics, Planning, and Control.

[6] Choset H.M., Hutchinson S., Lynch K.M., Kantor G., Burgard W., Kavraki L.E., Thrun S., Arkin R.C. (2005). Principles of Robot Motion: Theory, Algorithms, and Implementation.

[7] Karaman S., Frazzoli E. (2011). Sampling-based algorithms for optimal motion planning. Int. J. Robot. Res..

[8] Janson L., Schmerling E., Clark A., Pavone M. (2015). Fast marching tree: A fast marching sampling-based method for optimal motion planning in many dimensions. Int. J. Robot. Res..

[9] Gharbi M., Cortés J., Simeon T. A sampling-based path planner for dual-arm manipulation. Proceedings of the 2008 IEEE/ASME International Conference on Advanced Intelligent Mechatronics.

[10] Kuffner J.J., LaValle S.M. RRT-connect: An efficient approach to single-query path planning. Proceedings of the IEEE International Conference on Robotics and Automation.

[11] LaValle S.M., Kuffner J.J. (2001). Rapidly-exploring random trees: Progress and prospects. Algorithmic Comput. Robot. New Dir..

[12] Preda N., Manurung A., Lambercy O., Gassert R., Bonfè M. Motion planning for a multi-arm surgical robot using both sampling-based algorithms and motion primitives. Proceedings of the IEEE/RSJ International Conference on Intelligent Robots and Systems (IROS).

[13] Kurosu J., Yorozu A., Takahashi M. (2017). Simultaneous Dual-Arm Motion Planning for Minimizing Operation Time. Appl. Sci..

[14] Kavraki L.E., Latombe J.C., Motwani R., Raghavan P. (1995). Randomized Query Processing in Robot Path Planning. J. Comput. Syst. Sci..

[15] Hsu D., Latombe J.C., Kurniawati H. (2006). On the Probabilistic Foundations of Probabilistic Roadmap Planning. Int. J. Robot. Res..

[16] De Santis A., Albu-Schaffer A., Ott C., Siciliano B., Hirzinger G. The skeleton algorithm for self-collision avoidance of a humanoid manipulator. Proceedings of the IEEE/ASME international conference on advanced intelligent mechatronics.

[17] Dietrich A., Wimböck T., Täubig H., Albu-Schäffer A., Hirzinger G. Extensions to reactive self-collision avoidance for torque and position controlled humanoids. Proceedings of the IEEE International Conference on Robotics and Automation.

[18] Sugiura H., Gienger M., Janssen H., Goerick C. Real-Time Self Collision Avoidance for Humanoids by means of Nullspace Criteria and Task Intervals. Proceedings of the 6th IEEE-RAS International Conference on Humanoid Robots.

[19] Martínez C., Jiménez F. (2019). Implementation of a Potential Field-Based Decision-Making Algorithm on Autonomous Vehicles for Driving in Complex Environments. Sensors.

[20] Sichkar V.N. Reinforcement Learning Algorithms in Global Path Planning for Mobile Robot. Proceedings of the International Conference on Industrial Engineering, Applications and Manufacturing (ICIEAM).

[21] Wang C., Zhang Q., Tian Q., Li S., Wang X., Lane D., Petillot Y., Wang S. (2020). Learning Mobile Manipulation through Deep Reinforcement Learning. Sensors.

[22] Guo S., Zhang X., Zheng Y., Du Y. (2020). An Autonomous Path Planning Model for Unmanned Ships Based on Deep Reinforcement Learning. Sensors.

[23] Bae H., Kim G., Kim J., Qian D., Lee S. (2019). Multi-Robot Path Planning Method Using Reinforcement Learning. Appl. Sci..

[24] Gu S., Holly E., Lillicrap T., Levine S. Deep Reinforcement Learning for Robotic Manipulation with Asynchronous Off-Policy Updates. Proceedings of the IEEE International Conference on Robotics and Automation (ICRA).

[25] Liu C., Gao J., Bi Y., Shi X., Tian D. (2020). A Multitasking-Oriented Robot Arm Motion Planning Scheme Based on Deep Reinforcement Learning and Twin Synchro-Control. Sensors.

[26] Umay I., Fidan B., Melek W. An Integrated Task and Motion Planning Technique for Multi-Robot-Systems. Proceedings of the IEEE International Symposium on Robotic and Sensors Environments (ROSE).

[27] Haarnoja T., Zhou A., Abbeel P., Levine S. Soft Actor-Critic: Off-Policy Maximum Entropy Deep Reinforcement Learning with a Stochastic Actor. Proceedings of the International Conference on Machine Learning.

[28] Andrychowicz M., Wolski F., Ray A., Schneider J., Fong R., Welinder P., McGrew B., Tobin J., Pieter Abbeel O., Zaremba W. Hindsight Experience Replay. Proceedings of the 31st Conference on Neural Information Processing System.

[29] Chen J., Zhou Y., Gong J., Deng Y. An Improved Probabilistic Roadmap Algorithm with Potential Field Function for Path Planning of Quadrotor. Proceedings of the Chinese Control Conference (CCC).

[30] Kim M., Han D.K., Park J.H., Kim J.S. (2020). Motion Planning of Robot Manipulators for a Smoother Path Using a Twin Delayed Deep Deterministic Policy Gradient with Hindsight Experience Replay. Appl. Sci..

[31] Latombe J.C. (1991). Robot Motion Planning.

[32] Lozano-Perez (1983). Spatial Planning: A Configuration Space Approach. IEEE Trans. Comput..

[33] Laumond J.P.P. (1998). Robot Motion Planning and Control. Lecture Notes in Control and Information Sciences.

[34] Bergen G.V.D., Bergen G.J. (2003). Collision Detection in Interactive 3D Environments.

[35] Ericson C. (2004). Real-Time Collision Detection.

[36] Fares C., Hamam Y. Collision Detection for Rigid Bodies: A State of the Art Review. GraphiCon 2005. https://https://www.graphicon.org/html/2005/proceedings/papers/Fares.pdf.

[37] Puterman M.L. (1994). Markov Decision Processes: Discrete Stochastic Dynamic Programming.

[38] Sutton R.S., Barto A.G. (2018). Reinforcement Learning: An Introduction.

[39] Sutton R.S., McAllester D., Singh S., Mansour Y. Policy Gradient Methods for Reinforcement Learning with Function Approximation. Proceedings of the 12th International Conference on Neural Information Processing Systems.

[40] Silver D., Lever G., Heess N., Degris T., Wierstra D., Riedmiller M. Deterministic Policy Gradient Algorithms. Proceedings of the 31st International Conference on International Conference on Machine Learning.

[41] Lillicrap T.P., Hunt J.J., Pritzel A., Heess N., Erez T., Tassa Y., Silver D., Wierstra D. (2016). Continuous control with deep reinforcement learning. ICLR (Poster). arXiv.

[42] Abdolmaleki A., Springenberg J.T., Tassa Y., Munos R., Heess N., Riedmiller M. (2018). Maximum a Posteriori Policy Optimisation. arXiv.

[43] Barth-Maron G., Hoffman M.W., Budden D., Dabney W., Horgan D., Dhruva T., Muldal A., Heess N., Lillicrap T. (2018). Distributed Distributional Deterministic Policy Gradients. arXiv.

[44] Degris T., White M., Sutton R. (2012). Off-Policy Actor-Critic. arXiv.

[45] Mnih V., Kavukcuoglu K., Silver D., Rusu A.A., Veness J., Bellemare M.G., Graves A., Riedmiller M., Fidjeland A.K., Ostrovski G. (2015). Human-level control through deep reinforcement learning. Nature.

[46] Fujimoto S., Hoof H., Meger D. (2018). Addressing Function Approximation Error in Actor-Critic Methods. arXiv.

[47] Haarnoja T., Tang H., Abbeel P., Levine S. (2017). Reinforcement Learning with Deep Energy-Based Policies. arXiv.

[48] Hasselt H.V. (2010). Double Q-learning. Adv. Neural Inf. Process. Syst..

[49] Green P.J., Silverman B.W. (1993). Nonparametric Regression and Generalized Linear Models: a Roughness Penalty Approach.

